# Gut Microbiota Profiles in Children and Adolescents with Psychiatric Disorders

**DOI:** 10.3390/microorganisms10102009

**Published:** 2022-10-11

**Authors:** Marcela Soltysova, Aleksandra Tomova, Daniela Ostatnikova

**Affiliations:** 1 Academic Research Center for Autism, Institute of Physiology, Faculty of Medicine in Bratislava, Comenius University, 813 72 Bratislava, Slovakia; 2 Child Psychiatry Outpatient Care Unit, Zvolen Hospital, 960 01 Zvolen, Slovakia

**Keywords:** gut microbiota, dysbiosis, child psychiatry, psychiatric disorders in children

## Abstract

The aim of our work is to summarize the current state of knowledge on gut microbiota differences in children and adolescents with psychiatric disorders. To find the relevant articles, the PubMed, Web of Science, and Google Scholar databases were searched. Articles in English presenting original data and comparing the composition of gut microbiota in child psychiatric patients with gut microbiota in healthy children and adolescents were selected. Finally, we identified 55 articles eligible for our purpose. The majority of patients with autism spectrum disorders (ASD) were investigated. A smaller number of studies evaluating the gut microbiota in children and adolescents with attention-deficit/hyperactivity disorder (ADHD), Rett syndrome, anorexia nervosa, depressive disorder (DD), and tic disorders were found. The main findings of this research are discussed in our review, focusing on the age-related gut microbiota specificity for psychiatric disorders and the differences between individual diagnosis. To conclude, the gut microbiota in children and adolescents with psychiatric disorders is evidently different from that in controls. The most pronounced differences are seen in children with ASD, less in ADHD. Moreover, the changes are not identical to those in adult psychiatric patients, as *Ruminococcus, Turicibacter,* and Bilophila were increased in adults, and decreased in children with ASD, and *Parabacteroides* and *Alistipes* were more frequently represented in adults, but less frequently represented in children with depression. The available data suggest some genera have a different abundance in individual psychiatric disorders (e.g., *Bilophila*, *Bifidobacterium*, *Clostridium, Coprococcus, Faecalibacterium,* and *Ruminococcus*), suggesting their importance for the gut–brain axis. Other bacterial genera might be more important for the pathophysiology of specific disorder in children and adolescents, as *Akkermansia* and *Desulfovibrio* for ASD, or *Romboutsia* for DD. Based on the research findings, we assume that gut microbiota corrections have the potential to improve clinical symptoms in psychiatric patients.

## 1. Introduction

Studying the composition of gut microbiota in various medical conditions has extended to many different fields of medicine and science during the last two decades. First, gut microbiota was associated with somatic diseases. Later, an increasing number of articles describing a possible link between mental health conditions and gut dysbiosis were discussed. Unfortunately, there is still limited evidence of the contribution of gut dysbiosis to psychiatric disorders in children and adolescents.

Multiple signaling pathways of bidirectional communication between the gut and the brain have already been described [1]. Therefore, from the current state of knowledge, we can assume that gut dysbiosis may lead to an elevated risk of developing psychiatric disorders. Although some studies have provided evidence for the presence of bacterial DNA in human placenta and amniotic fluid, major bacterial colonization occurs at birth. The composition of the child gut microbiota depends on the delivery mode (vaginal or cesarean section), type of feeding of the newborn (breastfeeding or formula feeding), type of introduced solid food, and the use of antibiotics, probiotics, and other drugs and supplements by mother or child [2,3]. Furthermore, host genetics, dietary habits, gut physiology, hygiene level, stress, and physical activity influence the gut microbiota [2,4,5,6,7,8]. At the age of 3 years, the gut microbiota seems to be stabilized and resembles the composition of microbiota in an adult [8].

Remarkably, the human brain also develops intensively during the first years of life, and it seems that different environmental factors, including gut bacteria and their metabolites, influence its developmental milestones [9]. As reported in animal experiments, bacterial metabolites may cause morphological changes in different brain structures [10]. Research has proven their impact on neurogenesis, BDNF levels, and neurotransmitters (NTs) and NT precursors and their receptors [11]. Furthermore, some bacteria were found to stimulate the production of pro-inflammatory cytokines, modulate the immune system, change the activation of the hypothalamic–pituitary–adrenal (HPA) axis and vagal signaling and influence blood–brain barrier (BBB) formation [9,12,13].

Furthermore, short-chain fatty acids (SCFAs), particularly acetate, butyrate, and propionate, important products of bacterial fermentation in the large intestine, have various neuroactive properties. They are known to take part in neurotransmitter synthesis and release, mitochondrial and immune functions, and gastrointestinal physiology [14].

Gut bacteria can also produce NTs or can stimulate their production in the host organism, as summarized by several authors [1,15,16]. It is still not clear whether most of the NTs produced in the gut can cross the BBB; although, for example, circulating tryptophan influences serotonin synthesis in the brain after crossing the BBB [14]. Several animal studies using germ-free (GF) mice found lower serum levels of serotonin (5–HT), dopamine, and γ-aminobutyric acid (GABA), and altered metabolites and precursors levels of these neurotransmitters in the gut lumen or urine [10].

The aim of our work is to summarize the current knowledge about gut bacterial composition in children and adolescents with psychiatric disorders. First, we provide an overview of studies published before September 2022, as well as their most important characteristics. Second, we discuss the main results separately with respect to the diagnosis. Finally, we point out methodological limitations and emphasize the necessity of further investigations to obtain more comparable data, which will help to better elucidate possible links between gut bacteria and behavior.

## 2. Materials and Methods

Scientific databases (PubMed/MEDLINE, Web of Science, and Google Scholar) were searched using the key words: (gut bacteria OR microbiota OR intestinal microbiota OR microbiome) AND (obsessive OR OCD OR autism OR pervasive disorders OR ADHD OR hyperactivity OR schizophrenia OR psychotic disorder OR depression OR depressive OR anxiety OR addiction OR eating disorders OR anorexia OR bulimia OR neurodevelopmental disorders OR mental deficit OR behavior problem OR speech disorder OR tic OR Tourrette) AND (children OR child) NOT (mouse OR mice OR pigs OR zebrafish OR animal OR rat) in September 2022. From 734 results, the review articles and meta-analysis, book chapters, meeting abstracts, early access, and retracted articles and letters (#268) were eliminated. The remaining 466 articles were evaluated for our purposes and 41 articles met our inclusion criteria. Only articles in English presenting original data comparing the composition of gut microbiota in child and adolescent psychiatric patients (up to the age of twenty) with the gut microbiota in healthy children were included. We decided not to exclude the study where older participants were also included for the control group [17].

Studies not reporting on gut microbiota in psychiatric patients (#294) as well as genetic studies (#5), animal studies (#2), adult studies (#8), studies evaluating oral or duodenal microbiota (#7), mycobiota (#8) and urine metabolites (#13) were excluded. Furthermore, we eliminated studies evaluating the impact of one bacterial strain on clinical symptoms (#11), antibiotic/prebiotic/probiotic/synbiotic or dietary impact on psychiatric patients or healthy participants (#40), microbial transplantation studies (#11), studies comparing gut microbiota with the severity of the disease symptoms exclusively (#9), and studies not methodologically suitable for our purpose (#16). Another fourteen included articles were identified by additional searching. Overall, 55 studies were evaluated for our review.

## 3. Results

Fifty-five papers fulfilled our inclusion criteria. Forty-one studies included children with autism spectrum disorder (ASD). The other fifteen studies involved participants with attention-deficit/hyperactivity disorder (ADHD) (#7), Rett syndrome (#1), anorexia nervosa (AN) (#1), depressive disorder (MDD) (#3), and tic disorders (#2). The main characteristics of the included studies are summarized in Table 1.

Taken together, the gut microbiota of 4826 participants (3630 participants in ASD studies, 472 in ADHD, 79 in Rett syndrome, 39 in anorexia nervosa, 458 in depression, and 148 in tic disorder studies) was evaluated. From 4826 participant, 2617 were patients, and 2199 healthy volunteers and neurotypical siblings served as a control group. In the study of Thapa et al. (2021), ten psychiatric patients formed the control group along with healthy participants [18]. The trials were performed predominantly on boys, except for anorexia nervosa and Rett syndrome study, where only girl participants were included.

The age range of participants varied depending on the diagnosis (see Table 1).

In the reviewed studies, stool samples were used for analysis. The majority of samples were stored at −80 °C within 24 h. Storage at −20 °C was reported in two earlier published studies, and in five cases, this information was not provided or was unclear. In one case, a microbial preservation solution was used and samples were subsequently stored at room temperature [19]. 16S rRNA sequencing was used to evaluate gut bacterial composition in most cases. As seen in Table 1, the diagnostic tools used to evaluate patients and healthy controls differed according to diagnosis.

Previous research proved that the composition of the gut microbiota varies according to weight, diet, drugs, and comorbid diseases. About one-third of the studies in our review reported the body mass index (BMI) of participants. In approximately half of the studies, the authors evaluated diet habits; nevertheless, the methods of evaluation were often not reported or unclear. Thirty-two studies included information about gastrointestinal (GI) symptoms in their participants. In two studies, participants with GI symptoms were excluded. In forty-six cases, patients using antibiotics (ATB) or pre/probiotics before sample collection were excluded. For detailed information, see Table 1.

Differences in gut bacterial phyla and genera in psychiatric patients compared with healthy controls are summarized in Table 2 and Table 3, respectively. Only statistically significant results and reported nonsignificant differences are shown. The findings are discussed in detail below.

### 3.1. Gut Bacterial Profiles in Children with Autism Spectrum Disorder

Autism spectrum disorder is a lifelong neurodevelopmental disorder. Stereotyped and repetitive behavioral patterns, and a deficiency in communication and social interaction are its core symptoms [20]. The most frequent cooccurring mental health conditions in autism are ADHD, sleep–wake disorders, anxiety and depressive disorders, conduct disorders, bipolar disorders, schizophrenia, and obsessive-compulsive disorder (OCD) [21]. Furthermore, food selectivity, eating disorders, and gastrointestinal symptoms are frequently reported [22,23]. When compared with typically developing children, ASD patients are estimated to have a fivefold increased risk of developing a feeding problem (i.e., picky eating and severe food selectivity) and an approximately threefold increased risk of developing GI symptoms (e.g., diarrhea, constipation, abdominal pain/discomfort, bloating, or soiling) [22,23,24]. Many GI symptoms may remain undiscovered and be presented by more pronounced problematic behavioral manifestations (aggressivity, disrupted behavior, or self-injury) [24]. Even though the etiology of disturbed eating and GI symptoms in ASD patients remains unclear, these findings support the theory of dysbiosis in ASD patients.

Several researchers suggest that differences in microbial diversity between ADS patients and healthy controls may be associated with autistic symptoms, but the results from forty-one reviewed studies were not consistent. Lower alpha diversity (microbial diversity within the same group’s samples) in ASD patients was described in ten studies [25,26,27,28,29,30,31,32,33,34], while in thirteen cases, higher alpha diversity was found [19,35,36,37,38,39,40,41,42,43,44,45,46]. Another eleven studies did not reveal any significant differences in alpha diversity between ASD patients and healthy controls [47,48,49,50,51,52,53,54,55,56,57]. In the rest of the studies, information about alpha diversity differences were not provided [58,59,60,61,62,63,64]. Differences in beta diversity (community diversity between different group’s samples) were reported in fifteen studies [19,25,27,28,31,33,34,38,39,41,44,45,49,52,54].

As shown in Table 2, a number of studies reported significant differences in the abundance of specific bacterial phyla in ASD patients in comparison to healthy controls. However, consistent results were not obtained. Six studies revealed a decreased *Bacteroidetes/Firmicutes* ratio in ASD [30,34,41,49,54,62]. Contradictory findings were reported by the same number of authors [36,38,40,52,53,57], and in four other studies, no significant differences were observed [28,37,39,64]. A similar number of studies reported decreased, increased, or no specific difference in the abundance of *Bacteroidetes*, *Firmicutes*, and *Actinobacteria*. For *Fusobacteria* and *Verrucomicrobia*, mostly no specific differences were found. In most cases, levels of *Proteobacteria* were found to be increased or without specific differences (see Table 2). Only one out of thirteen studies bringing information about *Proteobacteria* reported decreased levels of this bacterial phylum [40]. However, while no statistically significant differences were found in nearly all other psychiatric diagnoses studied, ASD was clearly associated with gut microbiota differences at the phylum level.

Although many of the reviewed studies found differences in genera abundance in children with and without ASD, no consistent findings were observed for different bacterial genera except for *Dialister* and *Phasolarctobacterium* genera, which were found to be reduced in ASD patients in most of the reported cases (see Table 3). Higher abundances of *Bacteroides*, *Parabacteroides*, *Lactobacillus*, and *Clostridium*, and reduced abundances of *Bifidobacterium, Dialister*, *Faecalibacterium*, *Streptococcus*, and *Veillonella* were among the most frequently discussed findings. Lower amounts of *Prevotella* or certain *Prevotella species* in ASD patients were found in several studies [25,30,31,37]. In contrast, some authors found the opposite [33,40,42,55,57].

Several studies have also focused on searching for links between gut dysbiosis and autism severity. Finegold et al. (2010) found significantly higher amounts of several *Desulfovibrio* species and *Bacteroides vulgatus* in children with severe autism than in children with mild autism [35]. Tomova et al. (2015) also described a strong correlation between *Desulfovibrio* abundance and the severity of autism. The authors also found an increased level of *Clostridium* cluster I, but this finding did not reach statistical significance [62]. Other studies revealed higher *Firmicutes* abundance in severe ASD cases compared with mild autism cases [53] and elevated unidentified *Lachnospiraceae*, unidentified *Erysipelotrichaceae*, and decreased *Faecalibacterium* strains in children with severe ASD [39]. A weak correlation between *Bacteroides* spp. With the total score on the Social Responsiveness Scale (SRS) was described, but there was no significant correlation between *Bacteroides* spp. and *Bifidobacterium* spp. with the five subscales of the SRS [54]. A correlation between alpha diversity and the abundances of *Bacteroides, Faecalibacterium*, and *Oscillospira* and the Childhood Autism Rating Scale (CARS) total score was observed [32]. The opposite was found in the study of Xie et al. (2022) [56].

Several studies have looked for correlations between gut dysbiosis and GI symptoms in ASD children due to the high frequency of GI disorders in this population. GI symptoms in autistic children were associated with higher levels of *Clostridium histolyticum* (*Clostridia* cluster I and II) [59] or a lower amount of *Faecalibacterium prausnitzii* [25]. In the latter study, the authors linked this finding to an increased risk of gut inflammation [25]. However, in another study, levels of *Faecalibacterium prausnitzii* were found to be significantly higher in ASD patients, while levels of *B. longum* were decreased [36]. No difference in the abundance of *Faecalibacterium prausnitzii* was found by two other authors [55,61]. Furthermore, Strati et al. (2017) described a positive correlation between *Eischerichia/Shigella* and *Clostridium cluster XVIII* and GI symptoms in ASD children [49], while Rose et al. (2018) found increased *Bacteriodaceae*, *Lachnospiraceae*, *Ruminococcaceae*, and *Prevotellaceae* in ASD children with GI symptoms when compared with healthy children with GI symptoms. However, the authors did not reveal the same difference between autistic and healthy children, both without GI symptoms [63]. Chloroplast taxa were also found to be increased in autistic children. An even more pronounced difference was seen in the ASD with functional GI disorders (FGID) group compared with the ASD without FGID and neurotypical siblings. The authors discussed the possible link to chia seed consumption in these patients [48]. In the constipated ASD group, decreased bacterial species belonging to the genera *Bacteroides*, *Prevotella*, *Phascolarctobacterium*, and *Paraprevotella* and enriched *Fusobacterium*, *Barnesiella*, *Coprobacter*, and *Actinomycetaceae* were reported [27,30]. The SCFAs synthetizing genus *Roseburia* was proposed as a biomarker of an ASD patient with GI symptoms and this led to a question whether SCFAs producing bacteria preserve gut microbiota ecology [33]. On the other hand, significantly increased *Ruminococcus* in the ASD group without GI symptoms, according to the authors, corroborate the role of this bacterial genus in the improvement of the GI status of patients [33]. In contrast, Gondalia et al. (2012) failed to find any significant difference between gut bacteria in ASD patients and healthy controls, or differences in gut bacteria composition when comparing autistic children with and without GI dysfunction. Similarly, the authors found no link between microbiota composition and autism symptom severity [47]. It is worth mentioning that, in this study, stool samples were stored at −20 °C before analysis, which might have influenced the results. Another study revealed similar differences in the gut microbiota in “picky eaters” in both ASD and healthy children, and thus, the authors propose that certain differences in gut bacteria are associated with eating habits [65]. Interestingly, in the same study, other bacteria were found to be characteristic of the gut microbiota in children with ASD compared to controls [65]. In the recently published study of Wong et al. (2022), the abundances of genera *Fusicatenibacter*, *Dialister*, and *Phasolarctobacterium* were different in the ASD group with GI symptoms when compared with ASD, as well as healthy participants, both without GI symptoms [34]. Moreover, in the last-mentioned study, enriched *Turicibacter* was observed in the ASD group with GI symptoms. Based on previous knowledge, authors propose the role of *Turicibacter* in the pathophysiology of GI symptoms [34].

Neuroinflammation and neuroimmune abnormalities have been established in ASD as key factors in its development and maintenance [66]. In some of the reviewed studies, the levels of proinflammatory cytokines and markers of intestinal permeability were concurrently investigated. Although not statistically significant, levels of fecal TNFα were increased in children with autism, and a strong correlation between TNFα levels and GI symptoms was found [62], but, significantly increased levels of TNFα were also described [19,26]. Likewise, higher levels of TGFß were observed [19,26,44], as well as an increase in levels of IL-2 and IL-4 [19]. IL-4 was found to be negatively correlated with the CARS total score [19]. Furthermore, higher levels of serum IL-10, neurotensin peptide, and Sortilin1 (SORT-1), and increased fecal high-mobility group box protein 1 (HMGB1), were found in ASD patients [26]. HMGB1 is a multifunctional nuclear protein that can be released from cells in response to tissue damage or inflammation. A high level of circulating HMGB1 has been found in various autoimmune and inflammatory diseases, and fecal HMGB1 has been previously proposed as a biomarker of intestinal mucosal inflammation [67,68]. The amount of HMGB1 significantly correlated with the occurrence and severity of GI symptoms in ASD patients and was then proposed by authors as a biomarker to detect GI symptoms in these patients [26]. Levels of fecal calprotectin, another marker of intestinal inflammation, were previously found to be higher in ASD patients, and these results correlated with ASD symptomatology [69]. However, no significant differences in the levels of fecal calprotectin, serum IgA levels, or erythrocyte sedimentation rate between ASD patients and healthy children, as well as constipated and nonconstipated subjects in both groups, were found in another study [49]. Similarly, no significant differences of sIgA levels, but significant increase of zonulin and lysozyme was observed in the study of Vernocchi et al. (2022) [33]. In contrast, Adams et al. (2011) revealed a lower level of lysozyme, but this study failed to find significant differences in other possible markers of inflammation, such as lactoferrin, white blood cells, mucus, and secretory IgA [60]. Increased concentrations of IL-5, IL-15, and IL-17, and elevated markers of intestinal permeability zonulin-encoding genes, were associated with GI symptoms in ASD [63]. These findings suggest that children with ASD have disturbed immune signaling and intestinal permeability, and are consistent with previous findings about the relationship between enhanced gastrointestinal inflammation and GI symptoms in ASD [70], except for the study of Yitik Tonkaz et al. (2022), where authors did not support the role of gut bacteria in the etiology of ASD [64].

SCFAs (short-chain fatty acids), such as butyric, propionic, acetic, and valeric acids, are the main end-products of bacterial fermentation in the gut, and are also considered possible factors contributing to ASD symptoms. The main producers of butyrate belong to the *Firmicutes* families *Ruminococcaceae* (e.g., *Faecalibacterium prausnitzii*), *Lachnospiraceae* (e.g., *Roseburia* spp., *Eubacterium rectale, Coprococcus* spp.), *Erysipelotrichaceae*, and *Clostridiaceae* [71,72]. Propionate is produced by species of Bacteroidetes (e.g., *Bacteroides* spp., *Veillonella* spp., or *Blautia* spp.), while *Akkermansia municiphila* produces both propionate and acetate and *Bifidobacterium* spp. produce acetate and lactate [71,72]. SCFAs’ influence on gut motility, mucus production, integrity of gut epithelium, and anti-inflammatory effects have already been proven [14]. In the brain, SCFAs help to maintain the blood–brain barrier (BBB) integrity and, after crossing the BBB, act as neuroactive molecules. They have a proven anti-inflammatory effect and an effect on the maturation of microglia, but the precise mechanism remains to be elucidated [14]. Moreover, SCFAs have an impact on appetite and energetic homeostasis in the hypothalamus, influence the sleep cycle, stimulate the expression of BDNF and nerve growth factor (NGF), neurogenesis and neuronal proliferation, and help the growth of progenitor neurons. Butyrate stimulates growth hormone secretion in the hypophysis and promotes consolidation of memory [14]. SCFAs can influence the level of NT and neurotrophic factors [10,14]. It seems that the beneficial impact of SCFAs on health also depends on their concentration in organisms [73]. Negative effect of high levels of SCFAs was also observed [74].

Lower total SCFA levels in stool samples of children with autism were reported in two studies [42,60], while higher levels were reported in another study [54]. Furthermore, lower fecal acetate and butyrate [27,60] and lower propionate in autistic patients were described [60]. According to the latter mentioned authors, these results were strongly associated with GI symptoms and autism severity [60]. Faecal valerate was found to be higher in ASD [27]. Moreover, the gut microbiota was positively correlated with SCFAs. At the family level, *Acidobacteria* and *Actinomycetaceae* were correlated with valeric acid, while *Streptococcaceae*, *Peptostreptococcaceae*, *Lactobacillaceae*, *Clostridiaceae_I*, *Family_XIII*, and *Leuconostocaceae* had a positive correlation with butyrate. *Streptococcaceae* were highly correlated with propionic acid, and *Desulfovibrionaceae* were correlated with propionic as well as acetic acid [27]. Other studies have found higher concentrations of butyrate [36,50], acetate, and propionate [50] in children with autism, as well as higher abundance and higher effect of enzymes involved in butyrate production [36]. Berding and Donovan (2018) associated these findings with specific dietary patterns [50]. In the study by Carissimi et al. (2019), a lower abundance of *E. coli* was found. The authors suggested that elevated propionate in ASD patients might also be explained by its reduced degradation as a consequence of the *E. coli* drop, in addition to the enhanced production by *Clostridia* [26]. However, no significant differences in the levels of acetate, propionate, and butyrate, and no differences in relative concentrations of SCFAs, were described [25,54]. Although many studies suggest the possible role of SCFAs in ASD manifestations, more research is required to prove this possibility.

### 3.2. Gut Bacterial Profiles in Children and Adolescents with Attention-Deficit/Hyperactivity Disorder

Attention-deficit/hyperactivity disorder (ADHD) is a common neurodevelopmental disorder with the prevalence of approximately 7% among children and adolescents [75]. It is mainly characterized by symptoms of inattention, motor hyperactivity, and impulsivity [76]. ADHD is highly heritable, but several environmental factors, including gut bacteria, are believed to play a role in its development. To date, little is known about the composition of the gut microbiota, specifically in children and adolescents with ADHD. We evaluated seven studies that involved children and adolescents up the age of 20 years, but, for now, these studies did not bring any consistent conclusions.

Alpha diversity of intestinal microbiota between the ADHD and control groups did not differ significantly in five studies [17,77,78,79,80]. One study reported decreased alpha diversity in ADHD patients, and moreover, this result negatively correlated with hyperactivity symptoms [81]. However, it must be noted that, in this study, all patients were taking methylphenidate for more than one year, and this medication was discontinued only 48 h prior to sample collection, which might have influenced the results [82]. Wang and colleagues (2020) revealed the higher diversity indexes (Shannon and Chao) in ADHD [6]. In terms of beta diversity, only two studies described significant differences at the bacterial phylum level. More abundant *Actinobacteria*, less abundant *Firmicutes* [17], and increased *Fusobacteria* were observed in the ADHD group [6]. The remaining four studies failed to find differences in the abundance of dominant bacterial phyla (see Table 2).

At the genus level, certain significant differences were found. Decreased levels of *Faecalibacterium* [77,78], *Haemophilus* [79], *Prevotella*, *Parabacteroides* [81], *Lachnoclostridium, Sutterella*, *Dialister* [78], and *Veillonella* [77] were reported in the ADHD group. On the other hand, elevated amounts of *Agathobacter, Anaerostipes*, *Lachnospiraceae_UCG_010* [80], *Neisseria* [81], *Fusobacterium* [6], *Ruminococcaceae_UCG_004* [79], *Enterococcus*, and *Odoribacter* [77] were observed. The genus *Ruminococcaceae_UCG_004* was associated with inattention scores on the Conners Adult ADHD Rating Scales (CAARS) and Conners Teacher Rating Scale (CTRS) [79].

A lower abundance of *Faecalibacterium* was negatively associated with the total Conners’ Parent Rating Scale (CPRS) score and the hyperactivity index score [78]. Wan and colleagues (2020) hypothesized that *Faecalibacterium* dysregulation may cause changes in inflammatory cytokine levels, and therefore participate in ADHD pathogenesis [77]. Lower plasma TNFα levels in ADHD patients vs. healthy controls were found in one study [80], but no association between other cytokine levels and ADHD were observed [80]. An earlier published study described a significant increase in *Eggerthella*, *Alistipes*, *Odoribacter*, *Parabacteroides*, and *Bifidobacterium*, while the latter was associated with significantly enhanced predicted biosynthesis potential of a dopamine precursor (phenylalanine) in the gut microbiome of ADHD patients, which was linked to altered reward anticipation responses in the brain, a neural hallmark of ADHD [17]. Abnormal levels of *Odoribacter* and *Enterococcus* were previously associated with dysregulated neurotransmitter production [83,84]. Therefore, these bacteria may play a potential role in the development of ADHD [77]. Furthermore, differences in certain species of *Bacteroides*, *Sutterella*, *and Neisseria* in the ADHD group might, according to the authors, serve as possible biomarkers for ADHD, since these species were correlated with ADHD symptoms, such as hyperactivity and impulsivity [6,81].

### 3.3. Gut Bacterial Profiles in Children with Rett Syndrome

Rett syndrome (RTT) is a neurodevelopmental disorder caused by mutations in the methyl CpG binding protein 2 (MECP2) gene [85]. This condition predominantly affects females and is associated with intellectual disability and early neurological regression that severely affects motor, cognitive, and communication skills, leading to psychomotor delay and autistic features [86]. Moreover, 90% of patients suffer from gastrointestinal and nutritional problems that pose a significant medical burden for their caregivers [87], and in the context of gut microbiota research, dysbiosis may be one of the causes contributing to GI difficulties.

One study published to date involved children and adolescents with RTT exclusively [88]. Alpha diversity was decreased and beta diversity differed significantly between patients and healthy controls [88]. At the phylum level, an increase in Actinobacteria and a decrease in Bacteroides and Bacteroides/Firmicutes ratios were observed. Multiple bacterial genera were found to be different from healthy controls (see Table 3). Especially, a decrease in *Prevotella* and *Faecalibacterium*, which supports the hypothesis of the proinflammatory status of the gut microbiota in Rett syndrome.

### 3.4. Gut Bacterial Profiles in Children and Adolescents with Anorexia Nervosa

Anorexia nervosa (AN) is a severe psychiatric disorder with a high mortality rate and, in many cases, a life-long course. The prevalence of AN in Europe and the U.S.A. varies between 0.9% and 4% [89,90]. AN is characterized by a restrictive diet and low body weight. Excessive physical activity, purging, and the misuse of various drugs are frequent compensatory mechanisms that lead to subsequent weight loss [91]. Disturbed body image and enormous fear of weight gain complicate the treatment [91]. Many somatic, hormonal, and psychiatric comorbidities worsen its prognosis [89].

Little is known about the relationship between gut dysbiosis and adolescent AN. To date, only one study has investigated the gut microbiota in adolescents with anorexia nervosa exclusively [92]. This study described differences in beta diversity, but not in alpha diversity, of AN patients [92]. At the genus level, an increase in *Anaerostipes* and a reduction in *Romboutsia* were observed [92].

It seems that the abundance of different bacteria changes in the course of anorexia treatment. Alpha diversity and the abundance of *Firmicutes* phyla increased after weight recovery, while *Bacteroidetes* decreased. Furthermore, increasing levels of *Fusicatenibacter*, *Lachnospiraceae*, *Ruminococcaceae*, and *Faecalibacterium*, and a decrease in levels of *Bacteroides*, were observed in AN patients at discharge from the hospital when compared with findings at admission. However, *Romboutsia* and unclassified *Enterobacteriaceae* remained decreased [92]. In this study, the potential influence of bacteria on the duration of treatment was also evaluated. The authors found that a higher abundance of unclassified *Lachnospiraceae* was associated with a shorter duration of treatment, and they linked this finding with the anti-inflammatory effect of *Lachnospiraceae* [92].

### 3.5. Gut Bacterial Profiles in Adolescents with Depression

Major depressive disorder (MDD) is a serious public health problem with an increasing prevalence and a wide range of adverse consequences. Epidemiological studies estimate the lifetime prevalence at 11%, with a significant increase across adolescence, and a markedly greater increase in women than in men [93].

Several studies examining the role of intestinal microbiota in adult depression have already been published, while three of them evaluated its potential role in adolescent depression [18,94,95]. There are no consistent findings about alpha and beta diversity differences between patients with depression and healthy controls in these studies. Thapa et al. (2021) failed to find statistically significant differences in the abundance of the main bacterial phyla and genera in MDD patients [18]. In contrast, two other articles brought the evidence of several differences in gut bacteria between patients and healthy controls [94,95]. Among 26 significantly increased bacterial genera, Ling et al. (2022) identified eight bacterial taxa to be the biomarkers of childhood depression (*Prevotella*, *Bifidobacterium*, *Eischerichia/Shigella*, *Agathobacter, Gemmiger*, *Streptococcus, Collinsella*, *and Klebsiella)* from which *Streptococcus* was the best predictor for MDD in children [94]. *Bacteroides* and *Faecalibacterium* as the anti-inflammatory genera were found to be decreased in patients with depression, and *Faecalibacterium* was negatively correlated with levels of pro-inflammatory cytokines [94]. Previously published studies on adult depression found negative correlation between the abundance of *Bacteroides* and *Faecalibacterium* and the severity of depressive symptoms [96,97]. However, inconsistently with these findings, Zhou et al. (2022) described enriched populations of *Bacteroides* in children with depression [95]. Enriched *Eischerichia/Shigella* and *unclassified_f_Enterobacteriaceae* were positively correlated with anxiety and depressive scores in the Self-Rating Anxiety Scale (SAS) and Self-Rating Depression Scale (SDS) [95]. Likewise, the abundances of *Faecalibacterium* and *Bacteroides* were similar between the patients and the healthy control group in the study of Thapa et al. (2021) [18]. These authors also showed that neither MDD nor selective serotonin reuptake inhibitors (SSRIs) use were associated with differences in gut bacterial composition in older adolescents [18]. A study performed on healthy children indicated that psychosocial stress might modulate the composition of the gut microbiota, and the authors suggested evaluating parasympathetic activity in future research [98].

### 3.6. Gut Bacterial Profiles in Children with Tic Disorders

Tic disorder (TD) is another neurodevelopmental condition, including provisional and chronic motor or vocal tic disorders and Tourette syndrome. Many of these patients suffer from comorbid psychiatric conditions, such as ADHD, obsessive-compulsive disorder, or depression, which may have an impact on patients’ daily functioning [99,100]. Etiology is not fully known, but both genetic and environmental factors may play a role in pathogenesis [101].

Two studies published to date did not find the differences in alpha diversity of patient samples in comparison with HC [102,103]. Significant differences in beta diversity were found in the study of Wang et al. (2022) [102]. Xi et al. (2021) revealed higher abundances of certain bacterial species, such as *Bacteroides plebeius* and *Ruminococcus lactaris*, and lower abundances of *Prevotella stercorea* and *Streptococcus lutetiensis* in TD patients [103]. The authors also found correlations between tic severity and the abundance of *Klebsiella pneumoniae*, *Akkermansia muciniphila*, *Bacteroides* spp., *Bifidobacterium* spp., and *Eubacterium* spp. Moreover, study data indicate that treatment with dopamine receptor antagonists result in changes in the gut bacterial community [103]. In the other study, a significant reduction in *Bifidobacterium* genus was found in TD patients [102]. Authors suggest its possible role in TD by affecting the release of neurotransmitters, and speculate about the possible use of *Bifidobacterium* to treat mild or moderate symptoms of tic disorders. In the same study, a significant increase in *Prevotella* and *Odoribacter* was revealed in children with TD [102]. *Prevotella* is believed to have a pro-inflammatory effect [104], and increased inflammatory factors were found in TD [105]. Increased *Odoribacter* was also found in neuropsychiatric diseases such as pediatric acute onset neuropsychiatric syndrome (PANS) and pediatric autoimmune neuropsychiatric disorders associated with streptococcal infections (PANDAS) [83], and thus, this finding may likewise suggest the relationship with the etiopathogenesis of tic disorders [102].

**Table 1:** Main characteristics of studies reporting about gut microbiota in children and adolescents with psychiatric disorders.

Ref. No	Study	Year	Country	Sample Size	Gender % M/F	Mean Age in Years	Mean BMI	Diagnostic Tool Patients Controls		Sample Storage	Medication Use *	Dietary Questionnaire	Evaluation of GIT Symptoms
	**ASD + PDD − NOS**												
[19]	Cao et al.	2021	China	ASD: 45 HC: 41	ASD: 80/20 HC: 82.9/17.1	ASD: 6.8 HC: 5.2	ASD: 16.6 HC: 15.2	AMSE CARS CABS DSM-5	Psychiatric evaluation ASD psychometric tests	Microbial preservation solution was used, samples stored at room temperature	No ATB, probiotic or psychiatric medication prior sample collection	N/A	N/A
[25]	Kang et al.	2018	USA	ASD: 23 HC: 21	ASD: 95.7/4.3 HC: 71.4/28.6	ASD: 8.4 HC: 10.1	N/A	ATEC PDD-BI	N/A	−80 °C	No ATB or antifungal drugs 1 month prior sample collection	yes	6-GSI
[26]	Carissimi et al.	2019	Italy	ASD: 30 HC: 14	ASD: 96.7/3.3 HC: 50/50	Median age ASD: 3 HC: 10	N/A	DSM-5 ADOS-2 GMDS	N/A	−80 °C	No ATB, probiotic or prebiotic 2 weeks prior sample collection	N/A	GIH
[27]	Liu et al.	2019	China	ASD: 30 HC: 20	ASD: 83/17 HC: 80/20	ASD: 4.4 HC: 4.3	N/A	DSM-5 ICD-10	N/A	−80 °C	No ATB, probiotic, prebiotic or antifungal drugs 3 months prior sample collection	Participants with special diets excluded	6-GSI
[28]	Ma et al.	2019	China	ASD: 45 HC: 45	ASD: 86.7/13.3 HC: 86.7/13.3	ASD: 7.0 HC: 7.3	N/A	DSM-5 CARS	N/A	−80 °C	No ATB, probiotic or prebiotic 3 months prior sample collection	79-item food frequency questionnaire	N/A
[29]	Wang et al.	2019	China	ASD: 43 HC: 31	ASD: 83.7/16.3 HC: 54.8/45.2	ASD: 4.5 HC: 3.1	N/A	DSM-5	No psychiatric disorder	−80 °C	N/A	yes	Rome IV
[30]	Dan et al.	2020	China	ASD: 143 HC: 143	ASD: 90.9/9.1 HC: 88.8/11.2	ASD: 4.9 HC: 5.2	N/A	DSM-5	No serious psychiatric disorder	−80 °C	No ATB, probiotic or prebiotic 3 months prior sample collection, no use of AIDs or antioxidant drugs	N/A	Rome IV
[31]	Kang et al.	2013	USA	ASD: 20 HC: 20	ASD: 90/10 HC: 85/15	ASD: 6.7 HC: 8.3	N/A	ADOS ADI-R ATEC PDD-BI	N/A	−80 °C	No ATB or antifungal drugs 1 month prior sample collection, probiotic and nutrient use recorded	Special diets and seafood consumption recorded	6-GSI
[32]	Chen et al.	2021	China	ASD: 138 HC: 60	ASD: 84.8/15.2 HC: 45/55	ASD: 6.1 HC: 6.7	N/A	ADOS CARS DSM-5 WISC-R WPPSI	Gesell development scales	−80 °C	No ATB 1 month prior sample collection	N/A	GSI
[33]	Vernocchi et al.	2022	Italy	ASD: 41 HC: 35	ASD: 87.8/12.2 HC: 60/40	ASD: 6.5 HC: 8	ASD: 16.5 HC: 15.8	DSM-5 ADOS-2 ADI-R CBCL IQ/DQ tests	N/A	−80 °C	ATB, probiotic and psychiatric medication recorded	Specific diets recorded	Rome IV
[34]	Wong et al.	2022	China	ASD: 92 HC: 112	ASD: 100/0 HC: 100/0	ASD: 8.4 HC: 8.1	N/A	DSM-5 AQ-10 SDQ SCAS-P	AQ-10 SDQ SCAS-P	−80 °C	No ATB or regular probiotic use 1 month prior sample collection, use of psychiatric medication recorded	yes 7 day food record	R4PDQ
[35]	Finegold et al.	2010	USA	ASD: 33 SIB: 7 HC: 8	ASD: 72.7/27.3 SIB: 28.6/71.4 HC: 62.5/37.5	Age range 2–13	N/A	Authors’ evaluation of social, language, sensory and behaviour impairment	N/A	−80 °C	No probiotic or ATB 1 month prior sample collection	Special diets recorded	yes
[36]	Coretti et al.	2018	Italy	ASD: 11 HC: 14	ASD: 81.8/18.2 HC: 57.1/42.9	ASD: 1.5 HC: 1.5	N/A	DSM-5 ADOS-2 ADI-R GMDS VABS CARS	N/A	−80 °C	No ATB, probiotic, prebiotic or synbiotic 1 month prior sample collection	3 days diet diary	Rome III
[37]	Li et al.	2019	China	ASD: 59 HC: 30	ASD: 84.7/15.3 HC: 66.7/33.3	ASD: 4 HC: 5	N/A	DSM-5 ADOS ABC	N/A	−80 °C	No ATB, probiotic or prebiotic 3 months prior sample collection, no AIDs or antioxidant drugs	N/A	yes
[38]	Zhai et al.	2019	China	ASD: 78 HC: 58	ASD: 71.8/28.2 HC: 53.4/46.6	ASD: 5.0 HC: 4.9	N/A	DSM-4-TR ICD-10 ATEC	No history of ASD or neurological diseases	−80 °C	No ATB for 1 month prior sample collection, no probiotic or prebiotic	N/A	Participants with GI symptoms excluded
[39]	Ding et al.	2020	China	ASD: 77 HC: 50	ASD: 76.6/23.4 HC: 78/22	ASD: 1.6 HC: 1.8	N/A	DSM-5 CARS Social life ability scale, Gessel Develop. Schedules WISC	N/A	−80 °C	No ATB, probiotic or prebiotic 1 month prior sample collection	yes	N/A
[40]	Zou et al.	2020	China	ASD: 48 HC: 48	ASD: 79.2/20.8 HC: 50/50	ASD: 5 HC: 4	ASD: 17.4 HC: 16.3	DSM-4 ADI-R CGI-S ABC-I	No serious psychiatric disorder	−80 °C	No ATB or probiotic prior sample collection	N/A	N/A
[41]	Ye et al.	2021	China	ASD: 71 HC: 18	ASD: 100/0 HC: 100/0	ASD: 4.3 HC: 4.6	N/A	ABC DSM-5	ABC DSM-5	−80 °C	No ATB or probiotic 1 month prior sample collection, no AIDs or antioxidant drugs in HC group	N/A	N/A
[42]	De Angelis et al.	2013	Italy	ASD: 10 PDD: 10 SIB: 10	46.7/53.3 in all participants	Age range 4–10	N/A	ADOS ADI-R CARS	N/A	−80 °C	No ATB, probiotic or prebiotic 1 month prior sample collection	N/A	Participants with GI symptoms excluded
[43]	Niu et al.	2019	China	ASD: 114 HC: 40	ASD: 83.3/16.7 HC: 50/50	ASD: 4.5 HC: 4.2	N/A	DSM-5 ATEC	N/A	N/A	No ATB, probiotic or other GI treatment 1 month prior sample collection	N/A	yes
[44]	Zurita et al.	2020	Ecuador	ASD: 25 HC: 34	ASD: 96/4 HC: 91.4/8.6	ASD: 8.9 HC: 8.5	N/A	ADI-R	SCQ	−80 °C	No ATB or steroids 2 weeks prior sample collection	24-h dietary record	N/A
[45]	Fujishiro et al.	2022	Japan	ASD: 7 HC: 9	ASD: 71.4/28.6 HC: 66.7/33.3	ASD: 10.1 HC: 14.6	N/A	WISC-IV Denver-II ASSQ M-CHAT Paediatric and Psychological evaluation	WISC-IV Denver-II ASSQ M-CHAT Paediatrician and Psychological evaluation	−80 °C	N/A	yes	yes
[46]	Wan et al.	2022	China	ASD: 64 HC: 64	ASD: 82.8/17.2 HC: 84.4/15.6	Median age ASD: 2.5 HC: 2.3	N/A	DSM-4 DSM-5	SRS-2	N/A	No ATB, antifungal drugs, probiotic or prebiotic 2 months prior sample collection	3 day food record Food frequency questionnaire	N/A
[47]	Gondalia et al.	2012	Australia	ASD: 51 HC: 53	ASD: 82.4/17.6 HC: 35.8/64.2	Age range 2–12	N/A	Psychiatrist, psychologist evaluation CARS	N/A	−20 °C	No ATB or antifungal drugs 15 days prior sample collection, probiotic use recorded	N/A	yes
[48]	Son et al.	2015	USA	ASD: 59 SIB: 44	ASD: 88/12 SIB: 48/52	ASD: 10.3 SIB: 10.0	N/A	ADOS CBCL	CBCL	−80 °C	No ATB or probiotic 1 month prior sample collection	One-week food/calorie count diary Special diets recorded	QPGS-RIII SSC
[49]	Strati et al.	2017	Italy	ASD: 40 HC: 40	ASD: 77.5/22.5 HC: 70/30	ASD: 11.1 HC: 9.2	N/A	DSM-5 ADOS ABC CARS	N/A	−80 °C	No ATB, probiotic or prebiotic 3 months prior sample collection, no use of AIDs or antioxidant drugs	N/A	Rome III
[50]	Berding et al.	2018	USA	ASD: 26 HC: 32	ASD: 73.1/26.9 HC: 59.4/40.6	ASD: 4.1 HC: 4.8	ASD M: 16.8 ASD F: 15.1 HC M: 16.3 HC F: 16.8	PDDBI-SV	N/A	−80 °C	No ATB, probiotic or prebiotic 3 months prior sample collection, no routine medication	YAQ 3-day food diary Patients with special diet excluded	6-GSI Bristol stool chart
[51]	Pulikkan et al.	2018	India	ASD: 30 HC: 24	ASD: 93.3/6.7 † HC: 62.5/37.5	Median age ASD: 9.5 HC: 9.5	Median BMI ASD: 14.78 HC: 15.79	CARS INDT-ASD ISAA	Paediatric evaluation	−80 °C	No ATB, AIDs or antioxidant 1 month prior sample collection	7 days diet diary No gluten-free diet	yes
[52]	Zhang et al.	2018	China	ASD: 35 HC: 6	ASD: 82.9/17.1 HC: 83.3/16.7	ASD: 4.9 HC: 4.6	N/A	DSM-5	Medical evaluation Parent interview	−80 °C	No ATB, probiotic, prebiotic or APs 1 month prior sample collection	Participants with special diets excluded	N/A
[53]	Ahmed et al.	2020	Egypt	ASD: 41 SIB: 45 HC: 45	ASD: 68.3/31.7 SIB: 48.9/51.1 HC: 62.2/37.8	ASD: 5.6 SIB: 4.3 HC: 5.4	N/A	DSM-5 CARS	N/A	−80 °C	No probiotic before sample collection	yes	6-GSI
[54]	Ha et al.	2021	Korea	ASD: 54 HC: 38	ASD: 79.6/20.4 HC: 47.4/52.6	ASD: 7 HC: 6	N/A	DSM-5 ADOS-2 ADI-R SRS IQ tests	Psychiatric evaluation IQ tests	−80 °C	No ATB 3 months and probiotic for 15 days prior sample collection	N/A	N/A
[55]	Plaza-Díaz et al.	2019	Spain	ASD: 48 HC: 57	N/A	ASD: 1.8 HC: 2.1	ASD: 15.9 HC: 16.2	ADOS PDD-BI ADI-R Battelle test CARS DSM-5 ICD-10	N/A	−80 °C	No treatment of behavioural comorbidities	24 h diet recorded Food frequency questionnaire	N/A
[56]	Xie et al.	2022	China	ASD: 101 HC: 104	ASD: 85.1/14.9 HC: 76.9/22.1	ASD: 4.3 HC: 4.4	ASD: 16.3 HC: 15.7	DSM-5 CARS	N/A	−80 °C	No ATB, antifungal, AIDs, antioxidant, probiotic or prebiotic 1 month prior sample collection	Participants with special diets excluded	N/A
[57]	Chiappori et al.	2022	Italy	ASD: 6 HC: 6	ASD: 83.3/16.7 HC: 50/50	Age range: ASD: 6–17 HC: 10–20	N/A	DSM-5	N/A	−80 °C	N/A	N/A	N/A
[58]	Sun et al.	2019	China	ASD: 9 HC: 6	ASD: 88.9/11.1 HC: 66.7/33.3	Age range 3–12	N/A	ICD-11	N/A	−80 °C	N/A	N/A	yes
[59]	Parracho et al.	2005	UK	ASD: 58 SIB: 12 HC: 10	ASD: 82.8/17.2 SIB: 58.3/41.7 HC: 60/40	ASD: 7 SIB: 6 HC: 6	N/A	N/A	N/A	−20 °C	No probiotic or prebiotic in HC group for 6 months prior study, ATB usage recorded	yes	yes
[60]	Adams et al.	2011	USA	ASD: 58 HC: 39	ASD: 86.2/13.8 HC: 46.2/53.8	ASD: 6.9 HC: 7.7	N/A	Professional assessment ATEC	Parent report	N/A	No ATB or antifungal drugs 1 month prior sample collection, probiotic use recorded	Seafood and fish-oil consumption recorded	6-GSI
[61]	Wang et al.	2011	Australia	ASD: 23 SIB: 22 HC: 9	ASD: 91.3/8.7 SIB: 50/50 HC: 44.4/55.6	ASD: 5.1 SIB: 6 HC: 4.8	N/A	CARS DSM-IV	ASSQ	−80 °C	ATB and probiotic use recorded	Special diets recorded	FGID questionnaire
[62]	Tomova et al.	2015	Slovakia	ASD: 10 SIB: 9 HC: 10	ASD: 90/10 SIB: 77.8/22.2 HC: 100/0	Age range ASD: 2–9 SIB: 5–17 HC: 2–11	N/A	ICD-10 CARS ADI	Psychiatric evaluation Parent interview	−80 °C	All participants medication free	N/A	Parental questionnaire
[63]	Rose et al.	2018	USA	ASD: 50 HC: 41	ASD: 84/16 HC: 92.7/7.3	Age range 3–12	N/A	DSM-4 ADI-R ADOS ABC	SCQ MSEL VABS ABC	N/A	No ATB or antifungal drugs 1 month prior sample collection	Dietary changes recorded	GIH Rome III
[64]	Yitik Tonkaz et al.	2022	Turkey	ASD: 30 SIB: 30 HC: 30	ASD: 86.7/11.3 SIB: 53.3/46.7 HC: 43.3/56.7	ASD: 7.3 SIB: 9.4 HC: 8.5	ASD: 17.7 SIB: 17.5 HC: 17.1	Psychiatric evaluation DAWBA DSM-5 CARS	Psychiatric evaluation DAWBA	−80 °C	No ATB or antifungal drugs 2 months prior sample collection, no probiotic or prebiotic prior sample collection	Yes Participants with special diets excluded	GSI
	**ADHD**												
[6]	Wang et al.	2020	Taiwan	ADHD: 30 HC: 30	ADHD: 76.7/23.3 HC: 60/40	ADHD: 8.4 HC: 9.3	N/A	K-SADS-E SNAP-IV WISC ADHD-RS	K-SADS-E SNAP-IV WISC ADHD-RS	−80 °C	Patients using ATB or probiotic excluded	N/A Vegetarians excluded	N/A
[17]	Aarts et al.	2017	Netherlands	ADHD: 19 HC: 77	ADHD: 68.4/31.6 HC: 53.2/46.8	ADHD: 19.5 HC: 27.1	ADHD: 23.8 HC: 23.0	DSM-4 K-SADS	N/A	−80 °C	No ATB or other medication 1 month prior sample collection	N/A	N/A
[77]	Wan et al.	2020	China	ADHD: 17 HC: 17	ADHD: 82.3/17.7 HC: 76.5/23.5	Median age ADHD: 8 HC: 8	ADHD: 16.1 HC: 15.9	K-SADS CPRS	K-SADS CPRS	−80 °C	No probiotic 1 month prior sample collection N/A for ATB use	yes Participants with special diets excluded	N/A
[78]	Jiang et al.	2018	China	ADHD: 51 HC: 32	ADHD: 49/51 HC: 68/32	ADHD: 8.5 HC: 8.5	ADHD: 16.4 HC: 16.1	K-SADS-PL CPRS	Clinical interview CPRS	−80 °C	No ATB and probiotic for 2 month prior sample collection or concurrent use or history of ADHD drugs	yes Vegetarians excluded	N/A
[79]	Szopinska-Tokov et al.	2020	Netherlands	ADHD: 41 HC: 47	ADHD: 63/37 Ctrl: 49/51	ADHD: 20.2 HC: 20.5	Median BMI ADHD: 23 HC: 22	K-SADS CTRS CAARS	N/A	−80 °C	19 patients using ADHD medication	N/A	N/A
[80]	Wang et al.	2022	Taiwan	ADHD: 41 HC: 39	ADHD: 73.2/26.8 HC: 56.4/43.6	ADHD: 8 HC: 10	ADHD: 17.5 HC: 17.8	K-SADS-E WISC-IV Conners‘ CPT SNAP-IV	K-SADS-E	−80 °C	No AIDs, ATB or probiotic 1 month prior sample collection, no history of ADHD medication	Vegetarians excluded	N/A
[81]	Prehn-Kristensen et al.	2018	Germany	ADHD: 14 HC: 17	ADHD: 100/0 HC: 100/0	ADHD: 11.9 HC: 13.1	ADHD: 19.0 HC: 18.0	K-SADS-PL CBCL FBB-HKS	K-SADS-PL CBCL FBB-HKS	Not clear	10 patients using ADHD medication for more than 1 year, 9 of them discontinued 48h prior sample collection	yes	N/A
	**Rett syndrome**												
[88]	Strati et al.	2016	Italy	RTT: 50 HC: 29	RTT: 0/100 HC: N/A	RTT: 12 HC: 17	N/A	Genetic testing CSS	N/A	−80 °C	No ATB, probiotic or prebiotic 3 months prior sample collection	All participants under Mediterranean-based diet	Rome III
	**Anorexia Nervosa**												
[92]	Schulz et al.	2020	Germany	AN: 19 HC: 20	AN: 0/100 HC: 0/100	AN: 15.77 HC: 16.35	AN: 15.8 HC: 20.3	DSM-5 EDI BDI SCAS EDE	EDI BDI SCAS	−80 °C	No ATB or probiotic 1 month prior sample collection, other medication recorded	N/A	N/A
	**Depressive Disorder**												
[18]	Thapa et al.	2021	USA	MDD: 110 HC: 27 PC: 10	MDD: 35/65 HC: 63/37 PC: 57/43	MDD: 19.5 HC: 20.3 PC: 19.1	N/A	IDS, BDI-II BAI DSM-IV-TR A-LIFE	N/A	−80 °C	No ATB 6 months prior sample collection	N/A	N/A
[94]	Ling et al.	2022	China	MDD: 92 HC: 48	MDD: 45.7/54.3 HC: 45.8/54.2	MDD: 8.84 HC: 9.27	MDD: 21.9 HC: 21.3	HAMD DSM-5 CCMD-3	HAMD	−80 °C	No ATB, probiotic, prebiotic, synbiotic or psychiatric drugs 1 month prior sample collection	yes	N/A
[95]	Zhou et al.	2022	China	DD: 70 HC: 101	DD: 37.8/62.2 HC: 46.6/53.4	D: 13.7 HC: 13.5	D: 19.6 HC: 19.9	ICD-10 Mini-International Neuropsych. Interview SDS SAS	Clinical interview	−80 °C	No AIDs, ATB, probiotic, prebiotic or synbiotic 1 month prior sample collection	N/A	Bristol stool scale GSRS
	**Tic Disorder**												
[102]	Wang et al.	2022	China	TD: 28 HC 21	TD: 60.7/39.3 HC: 61.9/38.1	TD: 8.2 HC: 7.9	TD: 19.3 HC: 18.8	DSM-5 Expert Consensus on Diagnosis and Treatment of TD in China	No psychiatric disorder No physical illness	−80 °C	No GCs, ISs and AHs 15 days prior sample collection No ATB and probiotic 2 months prior sample collection	N/A	N/A
[103]	Xi et al.	2021	China	TD: 49 HC: 50	TD: 77.6/22.4 HC: 78/22	TD: 8.84 HC: 8.78	TD: 18.3 HC: matched	DSM-5 YGTSS	No psychiatric disorder	−80 °C	No ATB, probiotic, vitamins, IMs 1 month prior sample collection	N/A	GSI

A-LIFE—Adolescents Longitudinal Interval Follow-up Evaluation. ABC—Aberrant Behavior Checklist. ABC—Autism Behavior Checklist. ABC-I—Aberrant Behavior Checklist Irritability subscale score. ADHD-RS—ADHD Rating Scale. ADI-R—Autism Diagnostics Interview—Revised. ADOS—Autism Diagnostics Observation Schedule. AIDs—anti-inflammatory drugs. AHs—antihistamines. AMSE—Autism Mental Status Exam. APs—Antipsychotics. AQ-10—Autism Spectrum Quotient, 10-item parent report version. ASD—Autism Spectrum Disorder. ASSQ—Autism spectrum screening questionnaire. ATEC—Autism Treatment Evaluation Checklist. BAI—Beck Anxiety Inventory. BDI—Beck Depression Inventory-II. CAARS—Conners Adult ADHD Rating Scales. CABS—Clancy Autism Behavior Scale. CARS—Childhood Autism Rating Scale. CGI-S—Clinical Global Impression Severity of Illness scale. CBCL—Child Behavior Checklist. CCMD-3—Chinese Classification of Mental Disorder. Conners‘ CPT—Conners Continuous Performance Test. CPRS—Conners Parent Rating Scales. CSS—Clinical Severity Score. CTRS—Conners Teacher Rating Scale. DAWBA—Development and Well-Being Assessment. DSM-4—Diagnostic and Statistical Manual of Mental Disorders—4th Edition. DSM-4-TR—Diagnostic and Statistical Manual of Mental Disorders—4th Edition, Text Revision. DSM-5—Diagnostic and Statistical Manual of Mental Disorders—5th Edition. EDE—Eating Disorder Examination. EDE-Q—Eating Disorder Examination-Questionnaire. EDI—Eating Disorder Inventory 2. FBB-HKS—German ADHD rating scale for children. FGID questionnaire—Functional Gastrointestinal Disorder questionnaire. GCs—glucocorticoids. GDS—Gesell Developmental Scale. GHQ—Gastrointestinal Health Questionnaire. GIH—GI history survey. GMDS—Griffiths Mental Development Scales. GSI—Gastrointestinal Severity Index. GSRS—Gastrointestinal Symptom Rating Scale. HAMD—Hamilton Depression Scale. HC—non-related healthy controls. ICD-10—International Statistics Classification of Disease and Related Health Problems, 10th Revision. ICD-11—International Statistics Classification of Disease and Related Health Problems, 11th Revision. IDS—Inventory of Depressive Symptomatology. IMs—immunomodulators. INDT-ASD—INCLEN Diagnostic Tool for Autism Spectrum Disorder. ISAA—Indian Scale for Assessment of Autism. ISs—Immunosupressants. IQ/DQ—Intelligent Quotient and Developmental Quotient. K-SADS—Kiddie Schedule for Affective Disorders and Schizophrenia. K-SADS-PL—Kiddie SADS—Present and Lifetime Version. MSEL—Mullen Scales of Early Learning. NSAI—Non-steroidal anti-inflammatory drugs. PC—Psychiatric Controls. PDD-BI—Pervasive Developmental Disorder Behavior Inventory. PDDBI-SV—Pervasive Developmental Disorder Behavior Inventory Screening Version. PDD-NOS—Pervasive Developmental Disorder Not Otherwise Specified. QPGS—RIII—Pediatric Rome III Version. ROME III Diagnostic Questionnaire for the Paediatric Functional GI Disorders. R4PDQ—Rome IV Diagnostic Questionnaires for Paediatric Functional Gastrointestinal Disorders: Parent-report Form for Children. SAS—Self-Rating Anxiety Scale. SCAS—Spence Children’s Anxiety Scale. SDS—Self-Rating Depression Scale. SCQ—Social Communication Questionnaire. SIB—siblings. SNAP-IV—Swanson, Nolan, and Pelham Version IV Scale. SRS—Social Responsiveness Scale. SRS-2—Social Responsiveness Scale second edition. SSC—Simon Simplex Medical History. VABS—Vineland Adaptive Behavior Scales. WISC—Wechsler Intelligence Scale for Children. YAQ—Youth and Adolescence Food Frequency questionnaire. YGTSS—Yale Global Tic Severity Sc. † HC mostly consisted of sibling or blood relatives of ASD children. If period of medication use is not mentioned, this was not precised in the original article.

**Table 2:** Bacterial composition differences in children and adolescents with psychiatric disorders—phylum level.

	Autism + PDD − NOS																																									ADHD							RTT	AN	DD			TD	
**Ref. No**	[59]	[35]	[60]	[61]	[47]	[42]	[31]	[48]	[62]	[49]	[50]	[36]	[25]	[51]	[63]	[52]	[26]	[37]	[27]	[28]	[43]	[55]	[58]	[29]	[38]	[53]	[30]	[39]	[40]	[44]	[19]	[32]	[54]	[41]	[57]	[45]	[64]	[33]	[46]	[34]	[56]	[17]	[78]	[81]	[79]	[77]	[6]	[80]	[88]	[92]	[18]	[94]	[95]	[103]	[102]
Acidobacteria																																																							
Actinobacteria																																																							
Bacteroidetes																																																							
Cyanobacteria																																																							
Firmicutes																																																							
Fusobacteria																																																							
Proteobacteria																																																							
Verrucomicrobia																																																							
B/F Ratio																																																							

increased abundance of bacterial phyla in psychiatric patients vs healthy controls.decreased abundance of bacterial phyla in psychiatric patients vs healthy controls.reported non-significant differences.

**Table 3:** Bacterial composition differences in children and adolescents with psychiatric disorders—genus level.

	Autism + PDD − NOS																																									ADHD							RTT	AN	DD			TD	
**Ref. No**	[59]	[35]	[60]	[61]	[47]	[42]	[31]	[48]	[62]	[49]	[50]	[36]	[25]	[51]	[63]	[52]	[26]	[37]	[27]	[28]	[43]	[55]	[58]	[29]	[38]	[53]	[30]	[39]	[40]	[44]	[19]	[32]	[54]	[41]	[57]	[45]	[64]	[33]	[46]	[34]	[56]	[17]	[78]	[81]	[79]	[77]	[6]	[80]	[88]	[92]	[18]	[94]	[95]	[103]	[102]
Acetanaerobacterium																																																							
Acetivibrio																																																							
Acinetobacter																																																							
Actinomyces																																																							
Aeromonas																																																							
Agathobacter																																																							
Akkermansia																																																							
Alkaliflexus																																																							
Alkaliphilus																																																							
Alistipes																																																							
Anaerofilum																																																							
Anaerostipes																																																							
Anaerovorax																																																							
Bacillus																																																							
Bacteroides																																																							
Barnesiella																																																							
Bifidobacterium																																																							
Bilophila																																																							
Blautia																																																							
Butyricimonas																																																							
Butyrivibrio																																																							
Citrobacter																																																							
Chloroplast taxa																																																							
Chryseobacterium																																																							
Clostridiaceae_un																																																							
Clostridiales ‡																																																							
Clostridium																																																							
Clostridium cl. I																																																							
Clostridium cl. IV																																																							
Clostridium XI																																																							
Clostridium cl. XIVa																																																							
Collinsella																																																							
Coprobacillus																																																							
Coprobacter																																																							
Coprococcus																																																							
Coriobacteriaceae																																																							
Coriobacteriales_un																																																							
Corynebacterium																																																							
Desulfovibrio																																																							
Dialister																																																							
Dorea																																																							
Eggerthella																																																							
Eisenbergiella																																																							
Eischerichia																																																							
Eischerichia/Shigella																																																							
Enhydrobacter																																																							
Enterobacter																																																							
Enterococcus																																																							
Erysipelatoclostrium																																																							
Erysipelotrichaceae																																																							
Ethanoligenens																																																							
Eubacterium ‡																																																							
Faecalibacterium																																																							
Flavonifractor																																																							
Fusobacterium																																																							
Gemmiger																																																							
Haemophilus																																																							
Helcococcus																																																							
Hespellia																																																							
Klebsiella																																																							
Lactobacillus																																																							
Lactobacillus/Enterococcus																																																							
Lactococcus																																																							
Lachnobacterium																																																							
Lachnoclostridium																																																							
Lachnospira																																																							
Lachnospiraceae ‡																																																							
Leuconostoc																																																							
Megamonas																																																							
Megasphaera																																																							
Mitsuokella																																																							
Neisseria																																																							
Odoribacter																																																							
Oscilospira																																																							
Parabacteroides																																																							
Parasutterella																																																							
Paraprevotella																																																							
Parvimonas																																																							
Phascolarctobacterium																																																							
Porphyromonas																																																							
Prevotella ‡																																																							
Proteus																																																							
Providencia																																																							
Pseudomonas																																																							
Pseudoramibacter																																																							
Romboutsia																																																							
Roseburia																																																							
Ruminoclostridium ‡																																																							
Ruminococcus ‡																																																							
Ruminococcaceae ‡																																																							
Scardovia																																																							
Shigella																																																							
Staphylococcus																																																							
Streptococcus																																																							
Streptomyces																																																							
Subdoligranulum																																																							
Sutterella																																																							
Turicibacter																																																							
Tyzzerella sub4																																																							
Veillonella																																																							
Veillonellaceae																																																							
Weissella																																																							

increased abundance of bacterial phyla in psychiatric patients vs healthy controls.decreased abundance of bacterial phyla in psychiatric patients vs healthy controls.reported non-significant differences.mixed results according to different bacterial taxa. ‡ different strains included.

## 4. Discussion and Conclusions

The intestinal microbiota is comprised of a population of microorganisms more numerous than host cells, with several functions responsible for physiological processes in the entire human body. Furthermore, the gut–microbiota–brain axis displays a bidirectional communication that involves several main pathways along which interaction occurs. Disturbances of this communication in the form of dysbiosis are associated with various diseases, including psychiatric disorders, such as ASD, ADHD, depression, Rett syndrome, AN, and TD.

Although patients diagnosed with psychiatric disorders have been shown to suffer from GI symptoms such as constipation, diarrhea, reflux, vomiting, or abdominal pain, a conclusive link between these symptoms and disorders is yet to be drawn. Nevertheless, recently, a mounting number of investigations of gut microbiota in patients with psychiatric disorders suggest its important role for mental health. This study is focused on children and adolescents because the gut microbiota changes with age [2] and any differences in compared groups could be age-specific. For example, the Firmicutes/Bacteroidetes ratio has been shown to increase with age [106]. Comparing the gut microbiota in the groups targeted here, we found some common and some different characteristics in children compared with adults. In depression, *Faecalibacterium* had decreased abundance in adults [107], and in children [94], and *Streptococcus* genera were increased in both groups [94,108]. *Parabacteroides and Alistipes* had increased levels in adults [109], but decreased levels in children [94,95], while *Lactobacillus* and *Eggerthella* were elevated in adults but not changed in children [109].

For ASD, the science dedicates attention mostly to children, but very few articles about microbiota in ASD adults show similar decreases of *Fusobacteria* phyla abundance in both groups [110]. Interestingly, this was the only change found in adults compared to numerous changes in children’s microbiota. At the genera level, the level of Fusobacterium was shown to be decreased in both children and adults, contrarily to the abundance of some *Clostridium* strains, *Bacteroides*, and *Desulfovibrio*, which was increased in both. *Ruminococcus*, *Turicibacter*, and *Bilophila* were more abundant in adults, and less abundant in children [34,35,49,50,110,111].

Since animal models are widely used for studying and understanding psychiatric disorders in humans, it is also important to point out that some genera with increased abundance in individuals with ASD, specifically, *Bilophila*, *Clostridium*, *Dorea*, and *Lactobacillus*, revealed the same changes in mouse models of autism. Other genera with decreased abundance in autistic individuals, e.g., *Blautia*, show the same picture in animals, as reported in a recent review [112]. Many other differences in gut microbiota were found exclusively in humans (e.g., *Alistipes*, *Ruminococcus*) or exclusively in animal models (e.g., *Asaccharobacter*) [112].

Most of the articles included in our review comparing the gut microbiota of children and adolescents with different psychiatric disorders at the phyla level, found significant differences of bacterial abundance in ASD, sometimes in ADHD, and a few available studies about MDD, RTT, AN, and TD barely found any difference. Although the abundance of some bacterial phyla had contradictory changes in ASD patients, *Proteobacteria* was increased in almost all studies (except one) and *Verrucomicrobia* was decreased. This suggests the significant role of gut microbiota for ASD, supported also by changed alpha and beta diversity, which was not reported for ADHD and other disorders. In collected studies, some genera were observed to be more frequently represented in ASD as *Bacillus, Clostridiales,* and *Klebsiella*, and others as *Actinomyces, Bifidobacterium, Bilophila, Eischerichia, Faecalibacterium, Flavonifractor, Phascolarctobacterium, Veillonella* and *Weissella* were found to be less frequently represented. From these studies, it can be summarized that *Bifidobacteriales*, *Clostridiales*, *Faecalibacterium*, and *Ruminococcaceae* are associated with ADHD, as evidenced by an increase in *Clostridiales, Enterococcus, Bifidobacterium, Odoribacter*, and *Ruminococcaceae*, and a reduction in *Faecalibacterium, Haemophilus, Prevotella*, and *Lactobacillus*.

Taken together with up-to-date data, some genera are suggested to be important for the entire gut–brain axis, as they show similar changes in abundance in various psychiatric disorders (e.g., *Bilophila*, *Bifidobacterium*, *Clostridium, Coprococcus, Faecalibacterium,* and *Ruminococcus*). Other bacterial genera could be more important for a specific disorder in children and adolescents, such as *Akkermansia* and *Desulfovibrio* for ASD, or *Romboutsia* for MDD, and might be candidates as triggers or etiological agents of these disorders.

Although different studies found similar changes of some strains in a specific disorder, the abundance of other strains is inconsistent along the different studies. The reason behind this could be the limitations applied to studies and their interpretation, including: geographical, cultural, dietary, and demographic variability within the study populations, intake of medications, and methodological alterations. Furthermore, different disorders are age typical for different age groups. In spite of this, evidence suggests the microbiota–gut–brain axis’ involvement in psychiatric disorders and very complex relations between the brain and the microbiota.

Many external and environmental factors play a role in the manifestation of psychiatric disorders, and many factors shape the composition of gut microbiota, which makes research in this field very demanding. As this review demonstrated, numerous studies revealed that gut microbiota in children and adolescents with psychiatric disorders differ from that in the healthy population. Namely, studies on ASD, ADHD, Rett syndrome, AN, depression, and tic disorders have been performed to date. Current research aims to determine which gut microbiota differences are associated with concurrent symptoms and which might be connected to the pathophysiology of specific behavioral manifestations. However, independently of that, modulation of gut microbiota might be a feasible way to at least alleviate symptoms of various diseases, including psychiatric disorders, especially in children and adolescents.

Possible modification of gut bacterial composition using specific probiotics, prebiotics, or fecal microbiota transplantations for health improvement has been of great interest in recent years. Although the use of gut microbiota modulation is in perspective, the current state of knowledge is far from conclusive because of the research complexity and various limitations. For conclusions that will lead to clear recommendations, a higher number of study participants is required. Accordingly, geographical location and corresponding diet, and the use of various psychotropic drugs and nutritional supplements must also be considered. An age-related bacterial signature needs to be identified and taken into account. Therefore, our review focused on child patients with psychiatric disorders. In addition, microbial species other than bacteria, such as viruses, fungi, or protozoa, both in the lumen of the intestine and in the mucosa and other body environments (saliva, urine, or buccal mucosa), as well as microbial metabolites, should also be investigated to determine their impact on behavioral manifestations in psychiatric patients. Longitudinal studies should be conducted in the future to better understand the relationship between gut microbiota alterations and human behavior.

## Data Availability

Not applicable.
